# Whole exome sequencing in three families segregating a pediatric case of sarcoidosis

**DOI:** 10.1186/s12920-018-0338-x

**Published:** 2018-03-06

**Authors:** Alain Calender, Pierre Antoine Rollat Farnier, Adrien Buisson, Stéphane Pinson, Abderrazzaq Bentaher, Serge Lebecque, Harriet Corvol, Rola Abou Taam, Véronique Houdouin, Claire Bardel, Pascal Roy, Gilles Devouassoux, Vincent Cottin, Pascal Seve, Jean-François Bernaudin, Clarice X. Lim, Thomas Weichhart, Dominique Valeyre, Yves Pacheco, Annick Clement, Nadia Nathan, in the frame of GSF (Groupe Sarcoïdose France)

**Affiliations:** 1 Genetics Department, Hospices Civils de LYON (HCL), University Hospital, East Pathology Center, LYON, B-A3, 59 Bld Pinel, 69677 BRON Cedex, France; 2 Department of biostatistics, University Hospital, Hospices Civils de LYON (HCL), Lyon, France; 3 Inflammation & Immunity of the Respiratory Epithelium - EA7426 (PI3) – South Medical University Hospital – Lyon 1 Claude Bernard University, 165 Chemin du Grand Revoyet, 69310 Pierre-Bénite, France; 4 Cancer Research Center, INSERM U-1052, CNRS 5286, 69008 Lyon, France; 5 Pediatric pulmonology and Reference Center for rare lung diseases RespiRare, Hôpital Trousseau, AP-HP, INSERM UMR-S938, Sorbonne University, Paris, France; 6 Pediatric pulmonology and Reference Center for rare lung diseases RespiRare, Hôpital Necker, Paris, France; 7 Pediatric pulmonology and Reference Center for rare lung diseases RespiRare, Hôpital Robert Debré, INSERM U-1142, University Paris Diderot VII, Paris, France; 8 Department of Pulmonology, University Hospital, Hôpital Croix Rousse, Lyon, France; 9 Department of Pulmonology, University Hospital, Hôpital Louis Pradel, Lyon, France; 10 Department of Internal medicine, University Hospital, Hôpital Croix Rousse, Lyon, France; 11 Histology and Tumor Biology, ER2 UPMC, Hôpital Tenon, Paris, France; 12 Medical University of Vienna, Center for Pathobiochemistry and Genetics, Institute of Medical Genetics, Währinger Straße 10, 1090 Vienna, Austria; 13 EA2363, University Paris 13, COMUE Sorbonne-Paris-Cité, 74 rue Marcel Cachin, 93009 Bobigny, France; 14 Assistance Publique Hôpitaux de Paris, Department of Pulmonology, Avicenne University Hospital, 93009 Bobigny, France; 15 AP-HP Pediatric pulmonology and Reference Center for rare lung diseases RespiRare, Hôpital Trousseau, INSERM UMR-S933, Sorbonne University, Paris, France

**Keywords:** Sarcoidosis, Whole-exome sequencing (WES), Sarcoidosis, Candidate genes

## Abstract

**Background:**

Sarcoidosis (OMIM 181000) is a multi-systemic granulomatous disorder of unknown origin. Despite multiple genome-wide association (GWAS) studies, no major pathogenic pathways have been identified to date. To find out relevant sarcoidosis predisposing genes, we searched for de novo and recessive mutations in 3 young probands with sarcoidosis and their healthy parents using a whole-exome sequencing (WES) methodology.

**Methods:**

From the SARCFAM project based on a national network collecting familial cases of sarcoidosis, we selected three families (trios) in which a child, despite healthy parents, develop the disease before age 15 yr. Each trio was genotyped by WES (Illumina HiSEQ 2500) and we selected the gene variants segregating as 1) new mutations only occurring in affected children and 2) as recessive traits transmitted from each parents. The identified coding variants were compared between the three families. Allelic frequencies and in silico functional results were analyzed using ExAC, SIFT and Polyphenv2 databases. The clinical and genetic studies were registered by the ClinicalTrials.gov - Protocol Registration and Results System (PRS) (https://clinicaltrials.gov) receipt under the reference NCT02829853 and has been approved by the ethical committee (CPP LYON SUD EST – 2 – REF IRB 00009118 – September 21, 2016).

**Results:**

We identified 37 genes sharing coding variants occurring either as recessive mutations in at least 2 trios or de novo mutations in one of the three affected children. The genes were classified according to their potential roles in immunity related pathways: 9 to autophagy and intracellular trafficking, 6 to G-proteins regulation, 4 to T-cell activation, 4 to cell cycle and immune synapse, 2 to innate immunity. Ten of the 37 genes were studied in a bibliographic way to evaluate the functional link with sarcoidosis.

**Conclusions:**

Whole exome analysis of case-parent trios is useful for the identification of genes predisposing to complex genetic diseases as sarcoidosis. Our data identified 37 genes that could be putatively linked to a pediatric form of sarcoidosis in three trios. Our in-depth focus on 10 of these 37 genes may suggest that the formation of the characteristic lesion in sarcoidosis, granuloma, results from combined deficits in autophagy and intracellular trafficking (ex: Sec16A, AP5B1 and RREB1), G-proteins regulation (ex: OBSCN, CTTND2 and DNAH11), T-cell activation (ex: IDO2, IGSF3), mitosis and/or immune synapse (ex: SPICE1 and KNL1). The significance of these findings needs to be confirmed by functional tests on selected gene variants.

**Electronic supplementary material:**

The online version of this article (10.1186/s12920-018-0338-x) contains supplementary material, which is available to authorized users.

## Background

Sarcoidosis is an enigmatic multisystem disease characterized by the development and accumulation of granulomas, a compact collection of macrophages which have differentiated into epithelioid and multinucleated giant cells associated with lymphocytes [1]. The disease is considered to be the consequence of granulomatous reaction after exposure to various antigens and/or mineral particles in individuals with a susceptible genetic background [1, 2]. Sarcoidosis may be a chronic, benign or fatal disease affecting mainly lungs, skin, eyes and various other organs. This inflammatory disease develops before the age of 50 years and its incidence varies around the world with the highest frequencies being found in Northern European countries (10 to 40 per 100.000) [1]. In children, sarcoidosis is a very rare disease with an estimated prevalence of 0.4 to 1.2 per 100.000 [3]. A recent French study on a series of 41 children with sarcoidosis provided updated information on the initial presentation, the management and the follow-up of the disease [4]. It affects mainly children older than 10 years, while one third of them are younger. The disease is often severe with general symptoms at the forefront (asthenia, fever) and multiple organs involved at diagnosis in 85% of the children. The evolution is marked by frequent relapses and at risk to lung fibrosis despite extensive corticosteroid and immunosuppressive treatments [5]. The relative contribution of genetic and environmental factors is unknown but an 80-times increase in risk for monozygotic twins suggests that genetic factors might account for 50–60% of the disease susceptibility [6]. Sarcoidosis is commonly sporadic with familial presentation occurring in 3–5% of cases. The pedigree analysis suggests an autosomal dominant mode with incomplete penetrance. The French National SARCFAM study, based on a clinical network including 29 clinical centers, allowed the collection of clinical data and DNA samples of a cohort of 140 families with at least two first-degree relatives affected by sarcoidosis and rare situations of nuclear families with pediatric cases [7]. We gave priority consideration to the analysis of pediatric clinical situations in which the child with sarcoidosis has healthy parents, ie trios, expecting that new mutations and /or gene variants transmitted on a recessive pattern may highlight specific genetic pathways involved in the disease. Initially the genetic studies were focused on the genes previously identified by GWAS as putative pathogenic determinants in the risk of sarcoidosis. One of candidate gene, BTNL2, codes a cofactor of CD86, a regulator of T-cell activation, and a BTNL2 rs2076530 SNP splicing variant has been shown to induce an ≈ 2.0 fold increase in risk of occurrence of the disease [7, 8]. However, we have previously shown that this variant did not discriminate the sporadic and familial forms of the disease, thus suggesting BTNL2 is not a major gene predisposing to sarcoidosis [7]. GWAS studies in several series have identified SNP’s directly or indirectly implicated in innate immunity, such as HLADRB, CCDC88B, ANNEXINA11 (ANXA11), XAF1 and other candidate loci [9]. Granuloma formation is a process with hypothesized immune synapse abnormalities, suggesting new trials for treatment that target B and/or T cells [10]. A remarkable feature of sarcoidosis is the compartmentalization of CD4 (+) T helper 1 (Th1) lymphocytes and activated macrophages in the affected organs to initiate the formation and maintenance of granulomas [11]. Another subset of T-cells, Th17 effector CD4 (+), which mediate the crosstalk between immune cells and tissues, has been shown to participate in the progression of granulomas and in the fibrotic phase of the disease. A third subgroup of immune cells, the CD4 (+) CD25 (+) regulatory T cell (Treg) showed an abnormal expansion which may be responsible for the paradoxical suppression of the immune response to tuberculin observed in sarcoidosis [12]. Taken together, the immune pattern in sarcoidosis is close to that observed in other Th1/Th17 related diseases, as tuberculosis, leprosy, chronic beryllium disease (CBD), ulcerative colitis or Crohn disease but molecular signals that control granuloma genesis are largely undefined. A series of data obtained from animal models suggested a triggering of granuloma formation by mineral pollutants such as carbon nanotubes [13]. On the molecular side, several previous biochemical studies have shown abnormalities in pathways involving NFƙB [14], STAT1 [15], Gαi [16], and RIP2/IRAK [17], highlighting the difficulty of targeting a specific signalling pathway for therapeutic purposes. It was recently shown that the constitutive activation of mTORC1 in a mouse model deficient for TSC2 promotes granuloma formation through a metabolic reprogramming mediated by CDK4, suggesting a crucial role of the TSC1/TSC2 complex and mTor pathway in granulomatous diseases [18]. Nevertheless, identifying the genes which might be related to familial predisposition remains challenging as sarcoidosis is considered as a complex multifactorial disease involving a synergistic effect of many genes carrying either rare and more common variants. Our working hypothesis that we have tested here is that a comparative WES analysis of children affected early in life by a serious form of sarcoidosis and both their unaffected parents (trio analysis) may identify de novo and recessive genetic variants that could potentially point to pathways acting alone or in combination in disease development.

## Methods

### Patient recruitment, clinical evaluation, and sample collection

We recruited 3 trios, defined by a pediatric case of sarcoidosis and their unaffected parents. The clinical data were retrieved through the reference center for rare lung diseases (RespiRare) network and samples were collected in the frame of the SARCFAM project which involved 28 French university departments of internal medicine and/or pulmonology. The genetic project has ethical committee approval (CPP SUD EST – 2 – IRB 00009118 – September 21, 2016) and the clinical and genetic studies are registered by the ClinicalTrials.gov- Protocol Registration and Results System (PRRS) Receipt under the reference NCT02829853.

### Trio 1 (T1), reported in [3, 19]

An 8-year-old girl presented with massive and painful splenomegaly, deep pancytopenia, and mild systemic inflammation. Her parents were healthy and non-consanguineous. A bone marrow biopsy revealed epithelioid granulomas, leading to consider the diagnosis of a granulomatous disease. She had an increased plasma concentration of angiotensin converting enzyme (ACE) and bilateral hilar and mediastinal enlarged lymph nodes with several lung nodules on the lung CT scan. Pulmonary function tests showed a decreased dynamic lung compliance with normal blood gases and CO transfer. The bronchoalveolar lavage (BAL) showed an increased total cell count with 50% lymphocytes with a CD4/CD8 ratio of 5.4. No mutation of the *NOD2/CARD15* gene was detected. She was treated by monthly methylprednisolone pulses in association with daily oral prednisone therapy. Her general condition improved and, 6 months later, complete blood count was normal, splenomegaly, hilar lymphadenopathies and the pulmonary nodules had significantly decreased. During the following 7 years, she presented multiple relapses that remain corticosteroid-sensitive. A methotrexate treatment was started and she is currently under evaluation.

### Trio 2 (T2)

The diagnosis of sarcoidosis was made in a 6-year-old boy presenting with fever, weight-loss and tiredness. He also suffered from dyspnea, obstructive sleep snoring and apnea related to laryngeal and tonsil infiltration. Endoscopic examination revealed laryngeal infiltrates and pulmonary CT scan pulmonary infiltrates. Transbronchial biopsies allowed the identification of characteristic epithelioid granuloma and inflammatory related lesions. He was treated by 3 pulses of methylprednisolone followed by a 1 year treatment with oral prednisolone. The evolution was regressive with the absence of complications or symptomatic expression to date. Recent examinations by X-ray, CT-scan and pulmonary function tests were normal. His parents were healthy and a priori non-consanguineous. No mutation of the *NOD2/CARD15* gene was detected.

### Trio 3 (T3)

Sarcoidosis occurs in a young girl (age 5 yr) expressing a systemic disease with fever and pancytopenia, hepatomegaly and splenomegaly, mediastinal and peritoneal lymphadenopathies. Pulmonary CT-scan revealed extensive bilateral, hilar and mediastinal lymph node enlargement. Bronchoalveolar lavage (BAL) showed an increase of lymphocytes (50%) with more than 90% of CD4+ T-lymphocytes. Salivary glands and medullary biopsies allowed the characterization of granulomas with epithelioid and giant cells without necrosis. The patient was treated with methylprednisolone for 1 year with disease recurrence observed 8 months after the end of the treatment. The evolution affected many organs, lungs, liver, spleen and kidneys, confirmed by a renal biopsy showing similar epithelioid and giant cells rich granulomas, without necrosis. Corticoid therapy was initiated, associated to immunosuppressive protocol using mofetilmycophenolate. The treatment has been modified since 1 year with decreasing doses of the immunosuppressive agent and recently, the patient was considered to be in a remission phase. No mutation of the *NOD2/CARD15* gene was detected.

### Targeted exome sequencing

Genomic DNA was captured using Agilent in-solution enrichment methodology (SureSelect Human **Clinical Research Exome**, Agilent) with the supplied biotinylated oligonucleotides probes library (Human **Clinical Research Exome**, Agilent), followed by paired-end 75 bases massively parallel sequencing on Illumina HiSEQ 4000 [20]. Sequence capture, enrichment and elution were performed according to manufacturer’s instruction and protocols (SureSelect, Agilent) without modification except that library preparation was performed with NEBNext® Ultra kit (New England Biolabs®). For library preparation 600 ng of each genomic DNA was fragmented by sonication and purified to yield fragments of 150–200 bp. Paired-end adaptor oligonucleotides from the NEB kit were ligated on repaired, A-tailed fragments then purified and enriched by 8 PCR cycles. 1200 ng of these purified libraries were then hybridized to the SureSelect oligo probe capture library for 72 h. After hybridization, washing, and elution, the eluted fraction was PCR-amplified with 9 cycles, purified and quantified by QPCR to obtain sufficient DNA template for downstream applications. Each eluted-enriched DNA sample was then sequenced on an Illumina HiSEQ 4000 as paired-end 75b reads. Image analysis and base calling is performed using Illumina Real Time Analysis (RTA 2.1.3) with default parameters. Library preparation, exome capture, sequencing and data analysis have been done by IntegraGen SA (Evry, France).

### Bioinformatics

Two independent bioinformatics analyses were conducted, for the purpose of reducing the risk of missing a variant of interest. Both used the hg19 assembly version of the human genome.

First, the Integragen© pipeline was used. Reads were mapped using Elandv2e, and duplicates were removed. The variant call was performed using CASAVA1.8. Regions with low mappability (QVCutoff < 90) and variants with a weak quality (10 for SNVs, 20 for indels), were filtered out.Variants were annotated with population databases (1000G, ESP, ExAC, plus an Integragen© in-house database of exomes) and with score predictions (SIFT, Polyphen). Functional consequences of variants were predicted by Variant Effect Predictor. The familial analysis was done on each trio using Eris software (Integragen©).

Second, our in-house pipeline (PASS) was used that follows the recommendations of the Broad Institute (https://www.broadinstitute.org/gatk/guide/best-practices). Reads were trimmed with Trimmomatic, and aligned using BWA-MEM. Duplicates were marked using PicardTools, and the alignments were realigned and recalibrated using GATK. The variant call and genotyping were performed using GATK HapoltypeCaller (gVCF mode) and GenotypeGVCFs. Then, variants were recalibrated using GATK VariantRecalibrator. Variant annotation was done by snpEff/SnpSift, using both population (1000 Genomes, ExAC) and pathogenicity score (SIFT, Polyphenv2, Mutation Taster) databases. The familial analysis was performed with genoFilter (an in-house script). For this purpose, GATK CalculateGenotypePosteriors was first used with a pedigree file of the trio in order to reassign genotypes and genotype qualities. Only variants with balanced allele frequencies (maximum of imbalance of 0.35/0.65), and a genotype quality superior to 20 were considered.

### Statistical and functional evaluation of variants in silico

Gene sequences of the gene including known variations were downloaded from http://www.ensembl.org/ Homo_sapiens/Transcript/Variation_Transcript/. Predicted consequences of genetic variations were evaluated by using online bioinformatics tools such as SIFT [21], PolyPhen-2 [22], and EX_Skip [23]. The same information is compiled in ALAMUT© Visual software (from Interactive Biosoftware©) which allows us to know if the variants observed have already been described in recessive or dominant genetic diseases. A statistical analysis has been performed on these scores. VCF files were annotated with the dbNSFP v3.5 database [24]. CADD (Combined Annotation Dependent Depletion) [25], SIFT and POLYPHEN ranked scores were recovered for the selected de novo and recessive variants and for all the variants in the same genes and in the same patients, a mean value of the scores was considered when variants affect several transcripts. The different scores being non-normally distributed, their median was compared in the two groups (selected de novo and recessive variant vs all other variants in the same genes) by a non-parametric Wilcoxon/Mann-Whitney test. To identify which pathways the deleterious variant carrying genes might be enriched in an over-representation gene enrichment KEGG pathway analysis was performed on all the deleterious variant carrying genes identified from T1, T2 and T3 amongst total identified variant carrying genes using WebGestalt [26].

### Sanger sequencing for variants confirmation

Primer oligonucleotides for polymerase chain reaction and DNA sequencing were located within the reference sequence for the SPICE1 and CASC5 (Knl1) gene and surrounding the c.1912 T > G (SPICE1) and c.3853A > G (CASC5), respectively. For SPICE1 a 698 bp amplicon was produced and sequenced using primers 5′-CCATGGTGGGCTAATGAAATGA-3′ and 5′-GCCAAACATAAGCCATCTAGCC-3′.

For CASC5 a 726 bp amplicon was produced and sequenced using primers 5′-AGATCACTAGGAGTCACACAA-3′ and 5′ CTTCCTTCTCTAACAAAGGACA-3′. Agarose gel electrophoresis was used as a PCR quality control with size controls. The PCR products were then purified by Illustra™ ExoProStar™. The sequencing was performed by Big Dye terminator v1.1 after purification with BigDye® XTerminator™ Purification Kit. Sequence delineation and base calling used an automated fluorescent DNA sequencer (Applied Biosystems™, model 3130xl).

### Estimation of the inbreeding coefficient (f)

Inbreeding coefficients for each individual of T1, T2 and T3 were estimated by using the FEstim method [27] implemented in the FSuite software [28]. FEstim is a maximum-likelihood method that takes marker dependencies into account through a hidden Markov model. It gives more-specific information about an individual’s genome than the genealogy does, because it better reflects the true proportion of the individual’s genome that is autozygous. To obtain unbiased estimates of the inbreeding coefficient (f), a set of markers in minimal linkage disequilibrium should be used. We thus used the set of around 33,000 common polymorphisms in European population recommended by FSuite’s authors and drew 100 subsamples of around 3900 markers. The inbreeding coefficient was then estimated as the median value of the 100 estimates obtained on the different subsamples [28]. A likelihood ratio test is then performed in FEstim to identify whether individuals are inbred or not.

## Results

### Inbreeding coefficient (f) of affected children (c) in trios 1, 2 and 3

Inbreeding coefficients were respectively 0 (*p* = 0.975045) for T1c, 0.007 (*p* = 0.08312) for T2c and 0 for T3c (*p* = 0.97502). These data are suggestive of the absence of consanguinity in the three patients: the null hypothesis stating that the individuals are outbred is not rejected at the 5% level.

### WES sequencing and data selection

**Fig. 1:**
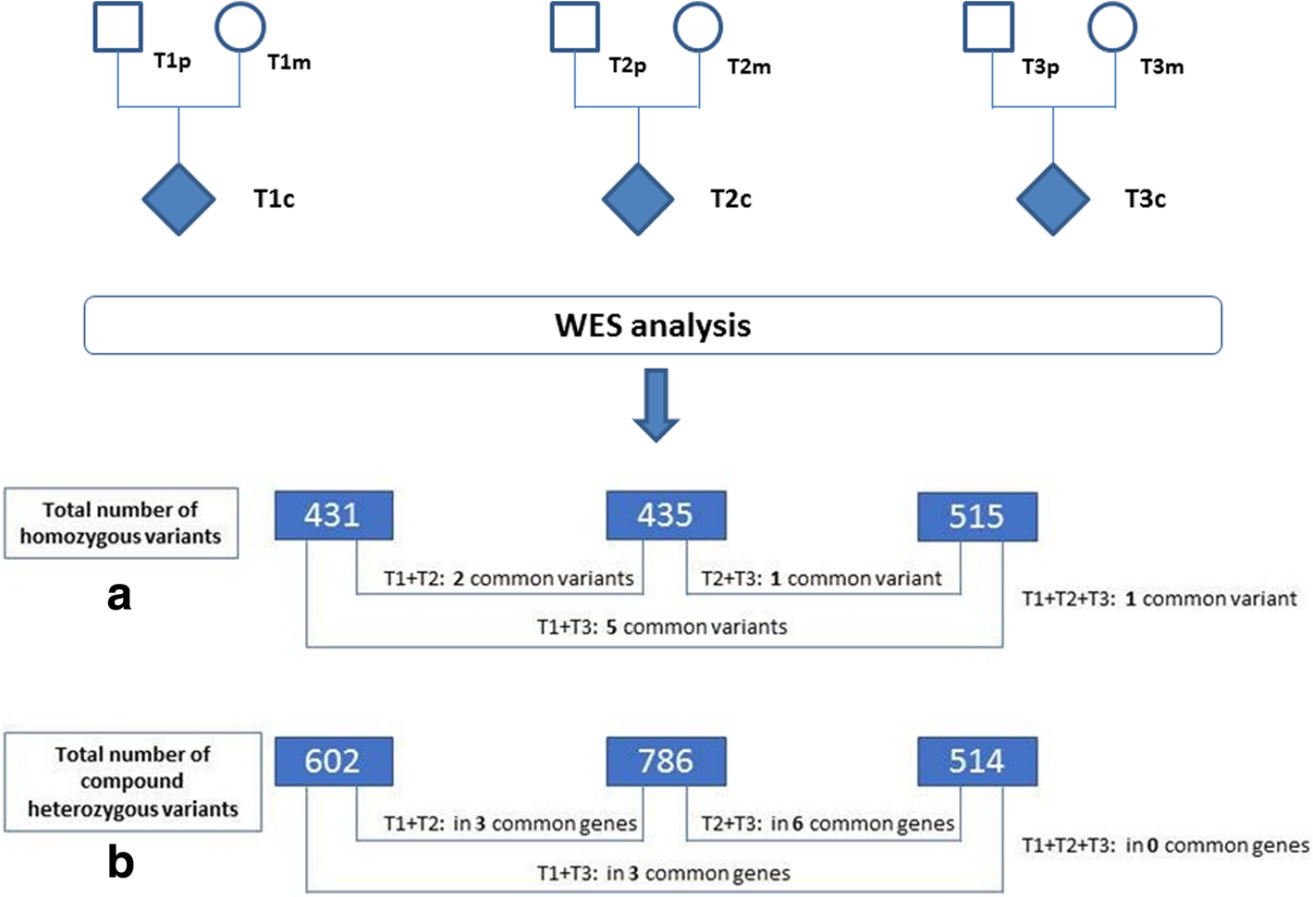
Flowchart of WES (Whole Exome Sequencing) analysis: T1, T2, T3 design the three nuclear families respectively, p (paternal) for the father, m (maternal) for the maternal and c for the affected child. The number in dark boxes indicate the total number of variants identified respectively in T1, T2 and T3 and inherited in affected children as (**a**) homozygous and (**b**) compound heterozygous variants. As mentioned in the text, we selected only those variants which were observed in at least two trios and putatively pathogenic as suggested by SIFT and/or POLYPHENv2 software

As shown in Fig. 1, the total number of variants inherited as homozygous recessive in the affected children represents 431 variants for trio 1 (404 missense, 3 frame-shifts, 6 in frame insertions, 14 splice site, and 4 start or stop codons lost), 435 variants for trio 2 (409 missense, 1 nonsense, 5 frame shift, 7 in frame deletion / insertion, and 2 stop codons lost), and 515 variants for trio 3 (478 missense, 4 nonsense, 8 frame shift, 12 in frame deletion / insertion, 12 splice site and 1 stop lost). We observed only 6 de novo mutations / variants, occurring in 6 genes and respectively 2 for T1 (IGSF3 and SPICE1), 2 for T2 (ZNF717 and CTNND2), and 2 for T3 (NPHS2 and PRSS55) (Table 1). We did not find recurrence of specific de novo mutations between two trios. Taking into account all values of minor allele frequency (MAF), we observed 9 variants in 9 different genes expressed as recessive traits in at least 2 trios, and respectively Sec16A and ADGRV1 (T1 + T2), RHBDL2, ZNF804A, AP5B1, TYR and CPAMD8 (T1 + T3), PRSS48 (T2 + T3) and OR11G2 (T1 + T2 + T3) (Additional file 1: Table S1). We observed also 12 genes sharing variations in at least two trios, but with different intragenic locations between the three patients (Additional file 2: Table S2). This concerns T1 + T2 (ASPN, WFDC3, CCT6B), T1 + T3 (OBSCN, SLC16A8), T2 + T3 (AFAP1, HSD17B4, DNAH11, CNGB1, MARCH10) and lastly T1 + T2 + T3 (RHBG, KNL1) for a total of 26 variants. We also took into account the possibility of composite heterozygocity which was observed in 11 genes, representing 44 variants and occurring in at least two trios: T1 + T2 (CMYA5, PCDHB16, RREB1), T1 + T3 (AIM1L, IDO2, KIR3DL1 and T2 + T3 (TDRD5, DNAH11, PIEZO1, FCGBP and PCNT) (Additional file 3**:** Table S3). By restricting MAFs to values less than 0.01, we identified 9 homozygous recessive variants occurring in a single trio (Table 2). This concerns five different genes for T1 (ZNF717, WNT2, GOG6, BEGAIN, NDUFV3), two for T2 (SHROOM1 and FMNL1) and two for T3 (DCP1B and CHRNA3).

**Table 1:** Possibly pathogenic de novo mutations observed in trios (T). We did not observe any variants common to at least two trios

Gene	Trios	Chr	Position	Variant	QUAL	Depth	Annotation	Reference	SIFT	Polyphen	EXaC
IGSF3	T1	1	117122285	I.F. INS	14,580	191	c.3122_3123insGGA p.Asp1040_Asp1041insGlu (NM_001542.3)	rs114915440	IN FRAME INS	IN FRAME INS	0.25
SPICE1	T1	3	113172543	SNP	3572	291	c.1912 T > G p.Ser638Ala (NM_144718.3)	nd	unknown	unknown	nd
ZNF717	T2	3	75781257	SNP	106,499	580	c.293A > C p.Gln98Pro (NM_001290210.1)	rs150224351	0.06	0	nd
CTNND2	T2	5	10981914	SNP	299	111	c.3388G > A p.Ala1130Thr (NM_001332.3)	nd	unknown	unknown	nd
NPHS2	T3	1	179526340	SNP	6106	530	c.560 T > C p.Met187Thr (NM_014625.3)	nd	unknown	unknown	nd
PRSS55	T3	8	10390473	SNP	3426	172	c.656G > C p.Trp219Ser (NM_198464.3)	nd	unknown	unknown	nd

Abbreviations: *nd* not defined, *Chr* chromosome, *SNP* single nucleotide polymorphism, *QUAL.* a quality parameter measuring the probability p that the observation of the variant is due to chance (for ex: QUAL = n, *p* = 1/n). *INS* insertion, *NM* NCBI reference sequence of mRNA, *ExAC* minor allele frequency as defined in Exome aggregation consortium

**Table 2:** Possibly pathogenic and rare (MAF < 0.01) recessive variants mutations observed in each single trio (T). We did not observe any variants common to at least two trios

Trios	Gene	Chr.	Position	Variant	QUAL	Depth	Annotation	Reference	SIFT	Polyphen	Function	EXaC
Recessive Variants – MAF < 0.01												
T1	ZNF717	3	75787726	SNP	221,913	1345	c.1048C > T p.Arg350Cys (NM_001128223.1)	nd	0.14	***0.689*** ^*^	Zinc finger protein Unknown	nd
T1	WNT2	7	116963030	SNP	7083	305	c.14 T > G p.Leu5Arg (NM_003391.2)	rs145839592	0.39	0.063	Wnt/ß catenin pathway	0.0011
T1	COG6	13	40254100	SNP	4954	228	c.624-3dupT frame shift (NM_020751.2)	rs397756552	**High impact**	**High impact **	Complex Oligomer Golgi complex	nd
T1	BEGAIN	14	101036074	SNP	20,030	273	c.-16 + 1G > C Splice donor (NM_020836.3)	rs10140554	**SPLICE DEFECT**	**SPLICE DEFECT**	Guanylate kinase-associated protein	nd
T1	NDUFV3	21	44317156	SNP	8515	341	c.168A > C p.Lys56Asn (NM_021075.3)	rs141922962	***0.01*** ^*^	***0.927*** ^*^	Mitochondrial complex I subunit	0.0051
T2	SHROOM1	5	132161699	SNP	13,112	130	c.134C > A p.Pro45Gln (NM_001172700.1)	rs143556262	***0.02*** ^*^	0.274	Regulator of the microtubule cytoskeleton	0.0065
T2	FMNL1	17	43320554	SNP	7318	194	c.2080G > A p.Ala694Thr (NM_005892.3)	rs76949926	1	0.121	Rac1-mediated cell migration and division	0.0019
T3	DCP1B	12	2062323	In frame INS	65,685	295	c.780_782dupGCA p.Gln261dup (NM_152640.3)	rs149912567	***nd*** ^*^	***nd*** ^*^	Decapping mRNA at the 5' end	0.00784
T3	CHRNA3	15	78913067	In frame DEL	27,072	105	c.67_69delCTG p.Leu23del (NM_000743.4)	rs66793222	***nd*** ^*^	***nd*** ^*^	nicotinic acetylcholine receptor	0.00138

Abbreviations: *nd* not defined, *Chr.* chromosome, *SNP* single nucleotide polymorphism, *QUAL.* a quality parameter measuring the probability p that the observation of the variant is due to chance (for ex: QUAL = n, *p* = 1/n). *INS* insertion, *DEL* deletion, *DUP* duplication, *NM* NCBI reference sequence of mRNA, *ExAC* minor allele frequency as defined in Exome aggregation consortium, SIFT and POLYPHEN scores are indicated in italic bold characters and an asterisk when considered as pathogenic in silico

### Statistical evaluation of prioritized variants

We performed a Wilcoxon/Mann-Whitney test to compare the deleterious effect of the prioritized variants, i.e., SIFT, PolyPhen, and CADD rank scores, against the other variants within the same gene [25]. The data showed no statistical differences for SIFT (dbNSFP_SIFT_rankscore: *p* = 0.4705) and POLYPHEN (dbNSFP_Polyphen2_HDIV_rankscore: *p* = 0.9322). We observed a statistically significant difference for the CADD score (dbNSFP_CADD_phred: *p* = 0.008468) (Additional file 4: Figure S1).

### Confirmation of variants and sanger sequencing

**Fig. 2:**
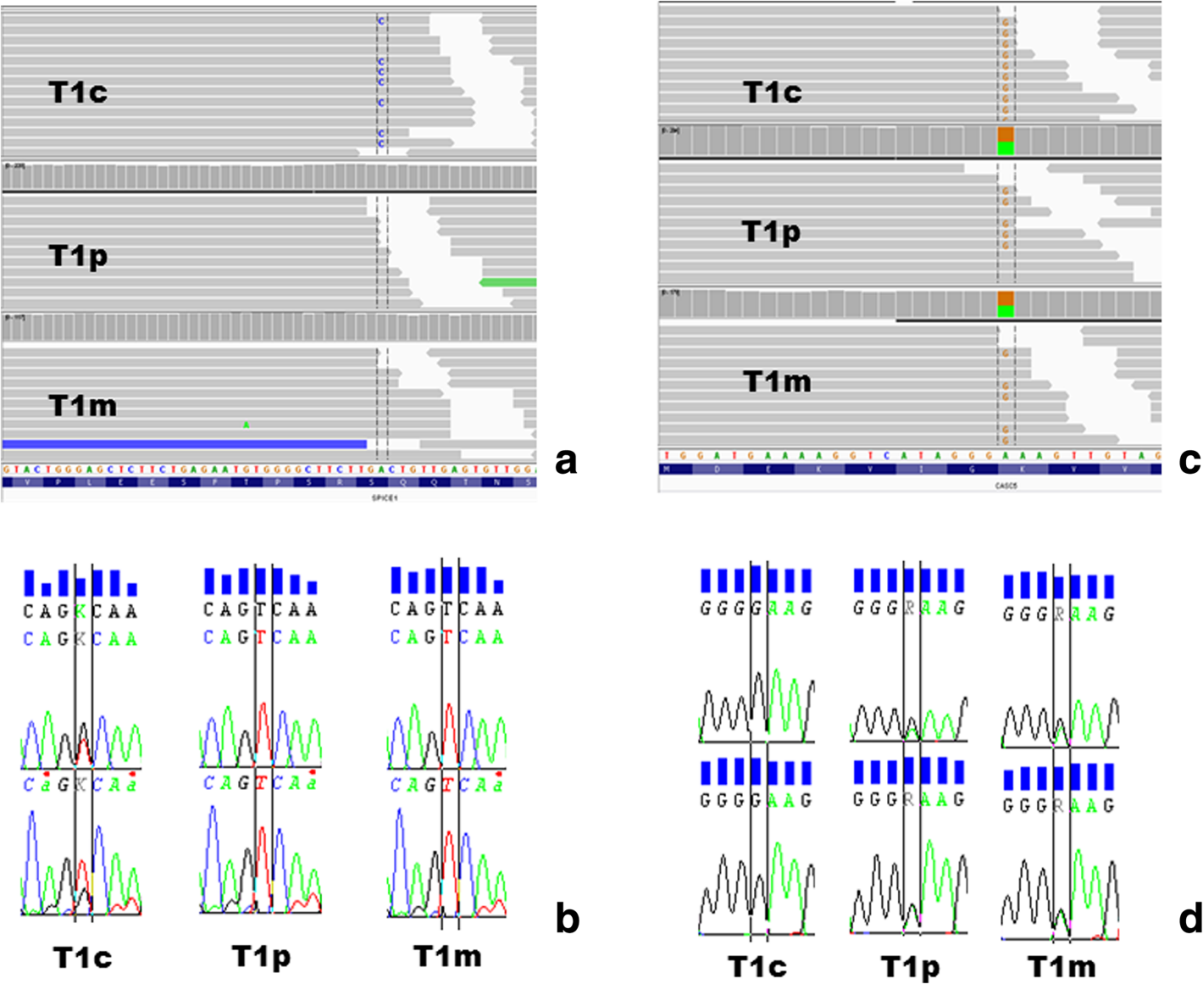
Parallel visualization of two gene variants identified in T1 by Integrative Genomics Viewer (IGV) (Broad Institute©) and Sanger Sequencing. **a** + **b** Confirmation of the de novo c.1912 T > G (c.1912A > C on reverse strand by WES) variant in the SPICE1 gene inherited by T1c (**a** + **b**), either on IGV (**a**) and Sanger sequence (**b**). **c** + **d** Confirmation of the homozygous c.2806A > G variant in the KNL1 (CASC5) gene inherited by T1c, either on IGV (**c**) and Sanger sequence (**d**). The IGV profiles show the absence of c.1912 T > G (SPICE1) in both T1p and T1 m parents (**a**) and the status of heterozygous carriers of T1p and T1 m for the c.2806A > G (KNL1) variant (**c**)

As these results were only descriptive and not associated to functional tests, all the gene variants were analyzed for their putative functional effect on the encoded protein with the SIFT and PolyPhen-2 online softwares databases. The quality (QUAL) of variants were assessed by a mean depth value of 349 [88–1012] and a mean quality score of 24,521 [299–183,916], suggesting that on average, the probability that the observed variants are due to a random occurrence is less than 0,000042. As shown in Fig. 2a the variants occurring in SPICE1 and KNL1 (CASC5) as de novo and recessive respectively, the IGV (Integrative Genomics Profile) confirmed the specificity of these observations according to the parental and child genomic profiles. Lastly, for some of these variants, we performed Sanger sequencing to confirm the presence of the variants found by WES (Fig. 2b).

### Functional interpretation of variants related to gene function

We based our in silico functional analysis on the putative pathogenicity of each variants, as suggested by SIFT and Polyphenv2 and then, an exhaustive search in PubMed (National Center for Biotechnology Information, U.S. National Library of Medicine) screening of published data linking a gene to immunity or related pathways. We performed an over-representation analysis of all deleterious variant carrying genes identified in T1, T2 and T3 probands amongst the list of total variant genes to find KEGG pathways that the deleterious genes might be enriched in. Interestingly, we found that these genes are enriched in pathways related to Antigen processing and presentation and that of the hematopoietic cell lineage (Additional file 5: Table S4). As shown in Table 3, among 37 genes, 10 (Sec16A, AP5B1, RREB-1, ASPN, RHBDL2, RHBG, KIR3DL1, TDRD5, MARCH10 and AFAP1) can be linked to autophagy and intracellular trafficking involving vesicles, Golgi apparatus and cytoskeleton, 6 genes (OBSCN, DNAH11, CTNND2, CNGB1 and NPHS2) play a direct or indirect role in G-protein regulation, 4 genes (IDO2, IGSF3, PIEZO1 and HSD17B4) may be related to T-cell activation, 6 genes (SPICE1, KNL1, CCT6B, PCNT, PCDHB16 and CMYA5) as regulation factors of transcription, cell cycle and immune synapse, and lastly, three (CPAMD8, WFDC3 and FCGBP) in innate immunity. Eight genes could not be classified. In order to summarize the most relevant data which implicate 4 major pathways that have been related to molecular / cellular defects in granuloma genesis, we presented the data for 10 of the 37 genes identified in this work and served as a basis for discussion of possible underlying pathological mechanisms related to sarcoidosis (Table 4).

**Table 3:** Functional summary on genes sharing variants in at least two trios

Functional pathways	Genes	Description / Interaction	Function	Related diseases	Add REF
Autophagy and trafficking	Sec16A	Endoplasmic Reticulum Export Factor	ULK1, ULK2 (autophagy initiation)	Familial axial spondyloarthritis	[35, 36, 66]
	AP5B1	Adaptator protein complex 5	AP membrane coat adaptor complex	Atopic march and psoriasis	[37, 67, 68]
	RREB-1	Ras responsive element binding 1	RalA and RalB GTPases, DNA transcriptional regulator	Unknown	[49, 69–71]
	ASPN	Asporin (PLAP-1), ligand of CD44	Regulation of TLR2 and TLR4 in macrophages	Osteoarthritis susceptibility	[72–75]
	RHBDL2	Rhomboid-like-2 serine protease	Regulation of EGFR and anoikis resistance, angiogenesis	Unknown	[76–78]
	RHBG	Rhesus-associated (Rh) glycoproteins	Ammonium transport in epithelial cells	Unknown	[79, 80]
	KIR3DL1	killer cell immunoglobulin like receptor	HLA interaction on NK and T-cells	Psoriasis, spondyloarthritis, Behçet	[81–83]
	TDRD5	Tudor domain containing 5	Cytoplasmic RNA processing	Spermatogenesis defects	[84, 85]
	MARCH10	Membrane-associated RING-CH 10	Microtubule-associated E3 ubiquitin ligase	Unknown	[86, 87]
	AFAP1	Actin filament associated protein 1	Actin-binding protein and a cSrc-activating protein	Unknown	[88–90]
G-proteins regulation	OBSCN	Obscurin	Rho-guanine nucleotide exchange factor	Dilated cardiomyopathies	[91, 92]
	DNAH11	Dynein axonemal heavy chain 11	Component of the ciliary structure	Primary ciliary dyskinesia	[93–95]
	CTNND2	δ-catenin/NPRAP/Neurojungin	Regulation of Rho-GTPases in cytoskeleton	Autism, complex human disorders^a^	[47, 96]
	CNGB1 (GARP)	Cyclic nucleotide gated channel beta 1	cGMP signaling, guanylate cyclase, expressed on T-reg	Retinitis pigmentosa	[97–100]
	ADGRV1	Adhesion G protein-coupled receptor V1	G-protein coupled receptor binding calcium	Usher syndrome type II^b^	[101, 102]
	NPHS2	Podocin (specific for podocytes)	Filtration barrier in mammalian kidney (TRPC channel)	Nephrotic syndromes	[103–105]
T-cell activation	IDO2	Indoleamine 2,3-dioxygenase 2	Acts for IDO1 dependent induction of T-regulatory cells	Colitis, arthritis	[52, 53, 106]
	IGSF3 (EWI-3)	Immunoglobulin superfamily member 3	Putative interaction with CD9 and CD81 (TPSPAN)	Unknown	[107–110]
	PIEZO1	Piezo type mechanosensitive ion channel 1	Mechanotransduction and regulation of cell morphology	Hereditary Xerocytosis	[111–113]
	HSD17B4	Hydroxysteroid 17-beta dehydrogenase 4	Beta-oxidation of very long chain fatty acids	PERRAULT syndrome^c^	[114, 115]
Cell cycle - immune synapse	SPICE1	Spindle and centriole associated protein 1	Regulation of centrioles elongation with CEP120 and CPAP	Unknown	[61, 116]
	KNL1 (CASC5)	Kinetochore scaffold 1	Stabilization of sister chromatids to microtubules	Unknown	[64, 117–119]
	CCT6B	Chaperonin containing TCP1 subunit 6B	Transcriptional response to stress in mitotic cells	Unknown	[120, 121]
	PCNT	Pericentrin, phosphorylated by PLK1	Ensure proper centrosome and mitotic spindle formation	Primordial dwarfism	[122–124]
	PCDHB16	Protocadherin beta 16	Cell-cell adhesion and intracellular signalling	Unknown	[125, 126]
	CMYA5 (TRIM76)	Cardiomyopathy associated 5, myospryn	Muscle target gene for the MEF2A transcription factor	Hypertrophic cardiomyopathy^d,e^	[127–130]
Innate immunity	CPAMD8	Alpha-2-macroglobulin domain containing 8	Up-regulated in immune stimulated cells	Anterior segment dysgenesis	[131, 132]
	WFDC3 (WAP14)	WAP four-disulfide core domain 3	Antimicrobial, immune, and tissue homoeostasis activities	Unknown	[133, 134]
	FCGBP	Fc fragment of IgG binding protein	Regulator of TGF-1-induced EM^f^ transition	Unknown	[135, 136]

Unclassified: OR11G2, PRSS48, ZNF804A, ZNF717, TYR, SLC16A8, PRSS55, AIM1L

Abbreviations of each gene name are detailed in the text

^a^the CTNND2 gene has been linked to a broad spectrum of human diseases, such as cancer, bipolar disorder, schizophrenia, autism, Cri-du-chat syndrome, myopia, cortical cataract-linked Alzheimer’s disease, and infectious diseases. ^b^Glycoprotein A repetitions predominant (GARP), a transmembrane protein containing leucine rich repeats (LRR), has been found to be highly expressed on the surface of activated T-reg cells. ^c^Patients with Usher syndrome type 2 present hearing loss and develop a visual impairment called Retinitis pigmentosa, a disease not observed in our cases, the c.6695A > G variant described in T1 + T2 being not described in this syndrome. ^d^PERRAULT syndrome is a rare recessive disease characterized by hearing loss, ovarian dysgenesis, intellectual disability and ataxia, a condition which is not observed in our cases, the c.1606 T > C (T2) and c.392G > A (T3) variants being not linked to this rare disease. ^e^the clinical screening of T1 and T2 affected children did not identify hypertrophic cardiomyopathy. ^f^EM: epithelial to mesenchymal transition

**Table 4:** Functional classification of 10 genes sharing putative pathogenic variants in at least two trios

Trios	Gene	Chr.	Position	Variant	QUAL	Depth	Annotation	Reference	SIFT	Polyphen	Alamut	EXaC
Autophagy and Intracellular Trafficking												
T1 + T2 (homoz)	Sec16A	9	139368953	SNP	28,526 23,786	602 430	c.3115C > T p.Arg1039Cys (NM_014866.1)	rs3812594	0.14	***0.689*** ^*^	Class3 Unknown Exon skip?	0.21
T1 + T3 (homoz)	AP5B1	11	65,547,333	SNP	15,898 27,369	187 219	c.631C > T p.Leu211Phe (NM_138368.4)	rs12146493	***0*** ^*^	***0.999*** ^*^	Class 3 Exon skip	0.29
T1 + T2 (c/het)	RREB1	6	7231843 7246998 7230680 7247344	SNP (T1p) SNP (T1 m) SNP (T2p) SNP (T2 m)	2748 6191 33,331 14,853	226 202 371 173	c.3511G > A p.Asp1171Asn (NM_001003698.3) c.4150G > A p.Gly1384Arg (NM_001003698.3) c.2348G > T p.Gly783Val (NM_001003698.3) c.4496C > A p.Ser1499Tyr (NM_001003698.3)	rs9379084 rs2281833 rs9502564 rs35742417	***0*** ^*^ 0.12 0.06 ***0.01*** ^*^	***0.999*** ^*^ 0 0.156 0.025	Class3 Unknown Exon skip? nd nd Class3 Unknown Exon skip?	0.11 0.27 0.44 0.14
G-Proteins Regulation												
T1 (homoz)	OBSCN	1	228505204	SNP	11,933	256	c.16472G > A p.Arg5491His (NM_001271223.2)	rs4653942	***0*** ^*^	***0.91*** ^*^	Class3 Unknown	0.26
T3 (homoz)			228494790	SNP	39,725	479	c.14986G > A p.Gly4996Arg (NM_001271223.2)	rs435776	***0.01*** ^*^	***0.939*** ^*^	Class 3 Unknown	0.41
T2 (de novo)	CTNND2	5	10981914	SNP	299	111	c.3388G > A p.Ala1130Thr (NM_001332.3)	nd	nd	nd	nd	nd
T2 + T3 (c/het)	DNAH11	7	21893993	SNP (T2p)	46,459	445	c.11122G > T p.Val3708Leu (NM_001277115.1	rs4722064	***0.02*** ^*^	***0.611*** ^*^	Class 3 Unknown	0.43
			21584693	SNP (T2 m)	1610	159	c.421G > T p.Asp141Tyr (NM_001277115.1)	rs72655969	***0*** ^*^	***0.601*** ^*^	Class 3 Unknown	0.012
			21,628,242	SNP (T3p)	20,259	553	c.1961C > G p.Ser654Cys (NM_001277115.1)	rs62441683	0.67	0.02	Class 3 Unknown	0.13
			21678643	SNP (T3 m)	19,593	850	c.4904A > G p.Asp1635Gly (NM_001277115.1)	rs17144835	***0*** ^*^	***0.783*** ^*^	Class 3 Unknown Exon skip?	0.045
T-Cell Activation and Immune Synapse												
T1 + T3 (c/het)	IDO2	8	39862881	SNP (T1p)	14,321	289	c.742C > T p.Arg248Trp (NM_194294.2)	rs10109853	***0*** ^*^	***1*** ^*^	Class 3 Unknown	0.48
			39873053	SNP (T1 m)	7771	184	c.1195G > A p.Ala399Thr (NM_194294.2)	rs72632016	0.57	0.024	Class 3 Unknown	0.06
			39872935	SNP (T3p)	21,240	451	c.1077 T > A p.Tyr359^*^ (NM_194294.2)	rs4503083	***STOP*** ^*^	***STOP*** ^*^	Truncating Polymorphism	0.226
			39862893	SNP (T3 m)	3510	154	c.754 T > A p.Ser252Thr (NM_194294.2)	rs35212142	***0.02*** ^*^	***0.444*** ^*^	Class 3 Unknown	0.02
T1 (de novo)	IGSF3	1	117122285	In frame INS	14,580	191	c.3122_3123insGGA p.Asp1040_1041insGlu (NM_001542.3)	rs114915440	IF INS	IF INS	In frame polymorphism	0.25
Mitosis and Immune Synapse												
T1 (de novo)	SPICE1	3	113172543	SNP	3572	291	c.1912 T > G p.Ser638Ala (NM_144718.3)	nd	nd	nd	Unknown	nd
T1 (homoz)	KNL1 (CASC5)	15	40916237	SNP	57,541	697	c.3853A > G p.Lys1285Glu (NM_170589.4)	rs17747633	***0.05*** ^*^	0.058	Class 3 Unknown Exon skip?	0.32
T2 (homoz)			40915190	SNP	183,916	368	c.2806A > G p.Arg936Gly (NM170589.4)	rs8040502	1	0	Class 3 Unknown	0.24
T3 (homoz)			40914177	SNP	66,997	297	c.1793 T > C p.Met598Thr (NM170589.4)	rs11858113	***0.01*** ^*^	0.006	Class3 Unkown	0.39

Abbreviations: *nd* not defined, *Chr.* chromosome, *SNP* single nucleotide polymorphism, *QUAL.* a quality parameter measuring the probability p that the observation of the variant is due to chance (for ex: QUAL = n, *p* = 1/n). *INS* insertion, *DEL* deletion, *DUP* duplication, *NM* NCBI reference sequence of mRNA, *ExAC* minor allele frequency as defined in Exome aggregation consortium, SIFT and POLYPHEN scores are indicated in italic bold characters and an asterisk when considered as pathogenic in silico

## Discussion

Sarcoidosis is a multifactorial disease that involves environmental pathogenic factors on a genetic background. Two sets of observations indicate a strong genetic heterogeneity. First, the rate of familial sarcoidosis remains low (3 to 5%) with most of the cases being a priori sporadic cases [7]. Second, a wide range of genetic variants have been previously identified by association studies in genes that may play a role in the pathogenesis of sarcoidosis, such as HLA-DP, BTNL2, Annexin A11, Toll-like receptors, CCDC88B (coiled-coil domain containing protein 88B), Ataxin/SH2B adapter protein 3, IL12B and NF-kappa-B p105 subunit [9, 29, 30]. The present work focuses on exceptional pediatric forms in terms of frequency and severity, with affected children having both parents healthy. We designed a WES genetic analysis on trios since a recent study suggested that for unsolved clinical exomes, extensive WES analysis of parent – offspring trios could identify likely contributory variant in 36% of cases [31]. Such an observation cannot be applied directly to sarcoidosis even if we might expect that comparing the exome of a patient and their healthy parents remains the most powerful strategy which has been used in many multifactorial diseases such in autism-spectrum disorders (ASD). As for ASD, both de novo and recessive variants must be considered as a powerful approach of relevant candidate genes [32]. Of the 37 genes identified in this work, 29 may be related directly or indirectly to pathways linked to immunity and related processes, such as autophagy, intracellular transport, T-cell activation and differentiation, cell cycle and immune synapse. The results obtained were selected according to strict quality criteria, including their reproduction in two independent analyzes, in-house pipeline PASS filtration, high analysis depth and suggestion of in silico pathogenicity in at least one of the software, SIFT or POLYPHENv2. Comparative statistical analysis of the selected variants versus other variants of the same gene showed no significant difference for the SIFT and POLYPHEN scores and a significant result for the CADD score (Additional file 4: Figure S1). The latter integrates several systems for analyzing the pathogenicity of variants and suggests that the variants observed belong to less than 10% of the most deleterious variants of the genome. However, the data should be analyzed with caution because many frequent polymorphisms in the genome have high SIFT and POLYPHEN scores and the non-parametric test performed in our study is likely to lack power given the small number of selected variants.

The variants and genes were classified in two ways:

First, according to the genetic transmission, we identified 6 de novo variants / mutations in 6 different genes without any overlap between the three affected children (Table 1). Except for IGSF3 and ZNF717, these variants were unknown in databases for SPICE1, CTNND2, NPHS2 and PRSS55 genes. All these heterozygous variants might induce pathogenic events and the function of genes was discussed later except those of ZNF717 and PRSS55 which remain unknown to date. Due to the very large number of variants observed by the WES technique in each of the trios, we then focused only on rare (MAF < 0.01) and recessive variations in each of the children of the trios (Table 2). This concerns 9 variants in 9 genes. A brief functional description for 4 of them (WNT2, COG6, FMNL1, DCP1B) was proposed in relation to immunity related process.

We then focused our attention on more common variants, all MAF values taken together, occurring in at least 2 trios and/or affecting a similar gene between at least two trios. They were classified as true homozygous recessive variants (Additional file 1**:** Table S1), or occurring in a common gene for at least two trios (Additional file 2**:** Table S2), and recessive heterozygous composites (Additional file 3**:** Table S3). This represents a total of 79 coding variants, 58 (73.4%) of them being putatively considered a pathogenic for the encoded protein by either SIFT and/or Polyphenv2. The integration in our work of common variants was absolutely necessary because sarcoidosis is probably a highly polygenic disease induced by the combined effect of rare pathogenic mutations and common polymorphisms in genes related to immunity.

In a second part, we have attempted a functional classification based on the fact that sarcoidosis as a multifactorial environmental and genetic disease may be related to defects in various subcellular, biochemical and immune targets susceptible to explain granuloma formation. We focused mainly on genes with recurring variants in at least two trios. The data are summarized for the whole series of genes in Table 3. We then detailed the functional discussion on 10 genes for which an interesting link has been identified in the bibliography and likely to suggest a link with the pathophysiology of the granuloma (Table 4). We were able to distinguish 4 functional categories

### Autophagy and intracellular vesicular trafficking

In contrast with Crohn’s disease, very little information is available on constitutive defects in autophagy for sarcoidosis. These processes are involved in the cellular response to metals and nanoparticles in experimental mice models of granuloma [33, 34]. For instance, Sec16A (affected in T1 + T2) is an interaction partner of ULK1 and ULK2 thought to be essential for initiating autophagy [35]. ULK-mediated phosphorylation of SEC16A regulated the assembly of endoplasmic reticulum (ER) exit sites and ER-to-Golgi trafficking of specific cargo. In frame deletions of SeC16A have been observed in familial forms of spondyloarthritis [36]. AP5B1 (Adaptator protein complex 5), recently implicated in atopic asthma, T1 and T3 share a p.Leu211Phe pathogenic variant [37]. Adaptator proteins sort cargo into vesicles for transport one membrane compartment of the cell to another and AP5 is considered as a candidate for a late endosomal coat, regulating endosomal sorting and vesicle budding from this compartment [38]. COG6, sharing a recessive frame shift mutation in T1 is a member of the conserved oligomeric Golgi (COG) complex which has been implicated in the regulation of endosome to trans-Golgi network retrograde trafficking [39]. Mutations in the COG genes have been shown to result in the mislocalization of some of the autophagy-related (Atg) proteins, which are critical components involved in autophagosome formation. Interestingly, single nucleotide polymorphisms of the COG6 gene have been recently shown as shared risk locus for rheumatoid arthritis and systemic lupus erythematosus [39]. Another interesting gene is RREB-1 (Ras-responsive element-binding protein 1), a zinc finger transcription factor, which is an effector of the RaIA/B small GTPases, acting as major regulator in exosome release, membrane trafficking, cytokinesis, cell shape and movement [40].

### G-proteins regulation

When in the GTP-bound active form, Rho GTPases transduce signals by binding to effector proteins involved in many cellular processes including regulation of the actin skeleton, microtubule dynamics, cell division, migration and adhesion [41]. Such a regulation occurs in various immune cells: monocyte/macrophage, T and B lymphocytes and neutrophils. The switch between the GDP inactive and GTP active of typical Rho GTPases is regulated by guanine nucleotide exchange factors (GEF) and GTPase-activating proteins (GAP). Rho GTPases are known to exert an effect on the cytoskeleton through NFƙB, a major player in inflammatory processes [41]. The present study identified genes encoding GTPases or GEF, such as OBSCN and CTNND2. OBSCN (OBSCURIN), mutated in T1 and T3, are giant cytoskeletal proteins with structural and regulatory roles on Rho-GEF activity and play a role in the maintenance of cell-cell adhesion and regulate epithelial to mesenchymal transition [42]. The T1 patient shares a rare and recessive variant in FMNL1, encoding one isoform of the formin proteins. Macrophages express multiple formins, including FMNL1, with potential to impact actin remodeling involved in migration [43]. Formins interact with GTP-bound Rho, Rac and Cdc42 GTPases and contribute to macrophage migration activity by stabilizing the lifespan of podosomes, a key actor of actin cytoskeleton dynamics during adhesion and migration within tissues [44, 45]. DCP1B (decapping enzyme 1), mutated in T3, interacts with DEF6, a Rho-family guanine nucleotide exchange factor, which contributes to the regulation of the spatiotemporal organization of components of T cell signaling pathways and Cdc42-dependent actin polymerization [46]. CTNND2 (Delta Catenin 2), affected by a de novo mutation in T2, is an adhesive junction-associated protein in the delta subfamily of the β-catenin superfamily and functions in Wnt signaling pathways to regulate gene expression and modulate Rho GTPases in cytoskeletal reorganization [47]. By regulating the activity of small GTPases and the disruption of E-cadherin based adherens junction, delta catenin 2 promotes cell migration and one might expect that such a process could play a role in macrophage fusion [48]. The observation of an uncommon recessive variant in the WNT2 gene (T1 patient) may be relevant of a pathogenic role of the canonical Wnt/ß-catenin signaling pathway in part of the inflammatory process occurring in sarcoidosis. Two other genes (DNAH11 and RREB1) present compound heterozygotes variants respectively in (T1 + T2) and (T2 + T3) and have been characterized as proteins interacting with small GTPases [49, 50].

### T-cell activation

The immune patterns and T-reg cells disturbances observed in sarcoidosis are similar to those observed in other Th1/Th17 related diseases [51]. IDO2 (indoleamine 2,3 dioxygenase) is a particularly interesting gene, as affected by deleterious variants both in T1 and T3 (Table 3). IDO2 is expressed in antigen-presenting cells (APCs), including dendritic cells (DCs) and B cells and deserves special attention by its function in T-cell regulation and differentiation in vitro [52, 53]. IDO2 may contribute to inflammatory processes by acting in monocyte-derived DCs to control regulatory T-reg cells, as a putative immunosuppressive factor. IDO1 and IDO2 are functional interacting partners and have been associated to various autoimmune states, such as colitis, arthritis and encephalomyelitis [53]. These conflicting results suggest both an immunosuppressive and a positive inflammatory influence [54]. Interestingly, the two variants observed respectively in T1 (p.Arg248Trp – rs10109853 – MAF = 0,353) and T3 (p.Tyr359* - rs4503083 – MAF = 0,226) are two common inactivating polymorphisms observed in humans [52]. The first, Arg248Trp, introduces a mutation at a catalytic residue that results in > 90% reduction in catalytic activity in in vitro assays [52, 55]. The second is a STOP codon at residue 359, leading to a truncated and catalytically inactive protein product. Based on allele frequencies it estimated that at least 25% of the population has a functionally inactive IDO2. The two affected children from T1 and T3 share a second allelic variant with a putative deleterious suggesting that expression of IDO2 may be dramatically affected in both cases. IDO2 is essential for the IDO1-dependent induction of T-regulatory cells [53]. It can be hypothesized that IDO2, in synergy with IDO1 is both a pro inflammatory factor acting in B lymphocytes to promote the cross talk between B and T cells, increasing the number of antibody-secreting cells, thus acting as an immunosuppressive agent by the control of T-reg cells. Inactivating mutations may be responsible for a decrease in the activity and / or number of regulatory T-cells, thereby inducing a pro inflammatory situation. These genes are already being studied as potential therapeutic targets. It has recently been shown that IDO2-specific monoclonal antibodies reduced auto reactive T and B cell activation and alleviated arthritis in two independent preclinical arthritis models [56]. A de novo variant has been observed in T1 for the IGSF3 gene, an in- frame insertion which high frequency (0,25) in control population. The function of IGSF3 may be related to the molecular background of immune synapse, involving members of the immunoglobulin (Ig) superfamily such as BTNL2 [29]. IGSF3 or EWI-3 belongs to a novel Ig subfamily containing a Glu-Trp- Ile (EWI) motif not seen in other Ig proteins and has a strong similarity with CD101, a T-cell marker notably expressed on T-REG with potent suppressor activity, and which expression regulates IL-17 production and disease activity in inflammatory bowel and rheumatoid diseases [57, 58].

### Cell cycle and immune synapse

At the crosstalk of innate immunity and the toxicity of environment-derived particles, a strong interest has been shown for a series of molecules that might play a role in promoting granuloma formation. However there is controversy about the molecular and cellular mechanisms generating polyploidy giant cells found in sarcoidosis granuloma. It was thought that these lesions were generated by a cell fusion mechanism; however evidence from other studies suggests that mitotic defects and DNA damage may generate polyploidy in macrophages [59]. Previous works have described elevated numbers of cells in the S + G2/ M phase in advanced forms of sarcoidosis, suggesting that such mitotic defects may affect different immune cell lineages [60]. In connection with these concepts, we were interested to observe an unknown de novo variant in T1, p.Ser638Ala in the SPICE1 gene which encodes a protein that localizes to spindle microtubules in mitosis and to centrioles throughout the cell cycle [61]. Together with centrosome proteins CEP120 or CPAP, SPICE regulates the elongation of centrioles, which serve as templates for the assembly of centrosome structures [62]. Interestingly, the centrosome plays also a crucial role in the assembly and function of immune synapse and cytokine production during inflammation [63]. We thus might expect that defects in the molecular interaction that impact on the docking of centrosome at the plasma membrane initiated by T-cell and APC interaction is a relevant process related to innate immunity. On the other hand, experimental depletion of SPICE1 causes severe mitotic defects [61]. KNL1 (CASC5), a kinetochore substrate of Aurora-B, share putative pathogenic variants in the three trios (Table 2). This gene has been involved in the stabilization of sister chromatids to microtubules during mitosis, maintenance of centromeres cohesion and regulation of mitotic checkpoint silencing [64, 65].

## Conclusions

Using a WES approach in pediatric forms of sarcoidosis and parent – offspring trios we have identified a panel of genes that led us to propose hypotheses about 4 functional pathways potentially mediating the underlying mechanism(s) of granuloma formation: autophagy related intracellular vesicular trafficking, G-protein signaling and Rho GTPases, immune synapse and T-cell activation, and lastly intimate mechanisms of cell cycle and mitosis regulation. An over-representation gene enrichment KEGG pathway analysis suggests that a significant number of genes selected belongs to pathways related to Antigen processing and presentation and that of the hematopoietic cell lineage We are aware that this study is of descriptive type and that some of the selected variants may have no direct role in the development of the disease. Nevertheless, these results fit with the fact that sarcoidosis is a multifactorial disease with a complex genetic background and agree the hypothesis of multiple and combined mechanisms in the genesis of the granuloma. Clinically, it is possible that the variable expressivity of the disease can be explained by a strong genetic heterogeneity, in particular between the different evolutionary forms. Further studies are awaited to compare gene variants identified in pediatric cases and autosomal dominant family forms. The characterization of a very broad panel of genes associated with the different clinical forms of sarcoidosis will ultimately not only define the relative risk of disease occurrence in families at risk, but also better characterize the evolutionary profiles and help to optimize therapeutic strategies.

## Additional files

Additional file 1: **Table S1.** Recessive variants found in at least two affected children of different trios. Possibly pathogenic recessive variants (polymorphisms) found by whole-exome -sequencing in at least two affected children of the trios (T). Chr., chromosome; SNP, single nucleotide polymorphism; QUAL., a quality parameter measuring the probability p that the observation of the variant is due to chance (for ex: QUAL = n, *p* = 1/n). As detailed in the text, Alamut® Visual integrates missense variant pathogenicity prediction tools and in silico study of variants’ effect on RNA splicing, allowing the assessment of their potential impact on splice junctions and splicing regulatory sequences. Alamut® Visual helped us also to exclude well known mutations identified in recessive diseases for those genes which have been related to known genetic diseases (as shown in Table 3). (DOCX 23 kb)

Additional file 2: **Table S2.** Recessive variants shared by a common gene in at least two different trios. Possibly pathogenic recessive variants observed at different positions for a single gene in at least two affected children of the trios (T). Abbreviations are the same as in Tables 1, 2 and Additional file 1**:** Table S1. (DOCX 31 kb)

Additional file 3: **Table S3.** Composite heterozygocity observed in a common gene in at least two different trios. Possibly pathogenic compound heterozygous variants (allelic heterogeneity) observed in different positions of a common gene in at least two trios. The origin of either the paternal and maternal allele was detailed for each variant. Abbreviations are the same as in Tables 1, 2, Additional files 1 and 2**:** Tables S1 and S2. (DOCX 51 kb)

Additional file 4: **Figure S1.** CADD scoring of prioritized variants versus other variants in the selected genes. (PDF 71 kb)

Additional file 5: **Table S4.** Over-representation gene enrichment KEGG pathway analysis was performed on all the deleterious variant carrying genes. Data of the over-representation gene enrichment KEGG pathway analysis was performed on all the deleterious variant carrying genes in trios 1, 2 and 3. (XLSX 9 kb)

Additional file 6: All recessive variants identified in cases T1, T2 and T3. The de novo dominant variants have been fully described in the manuscript. The datasets of cases T1, T2 and T3 extracted from VCF (Variant call Format) and extracted in XLS file. (XLSX 368 kb)

## Abbreviations

1000G: 1000 genomes
BTNL2: Butyrophilin like 2
CCDC88B: Coiled-coil domain containing protein 88b
CPP: Comité de Protection des Personnes
CT: Computed tomography
ESP: Exome sequencing project
ExAC: Exome aggregation consortium
GTP/GDP: guanine tri/diphosphate
GWAS: Genome wide association studies
MAF: Minr allele frequency
mTor: Mammalian target of rapamycin
NOD2: Nucleotide binding oligomerization domain containing 2
Polyphenv2: Polymorphism Phenotyping v2
QPCR: Quantitative polymerase chain reaction
SARCFAM: Familial sarcoidosis
SIFT: Sorting Intolerant From Tolerant
SNP: Single nucleotide polymorphism
STAT1: Signal transducer and activator of transcription 1
TSC1/2: Tuberous sclerosis
XAF1: X inhibitor of apoptosis associated factor 1
