# Supplementary material for: Whole exome sequencing in three families segregating a pediatric case of sarcoidosis
Source: BMC Med Genomics. 2018 Mar 6;11:23. doi: 10.1186/s12920-018-0338-x (PMC5839022; doi:10.1186/s12920-018-0338-x)
Supplement: Supplementary file 1 — Table S1. Recessive variants found in at least two affected children of different trios. Possibly pathogenic recessive variants (polymorphisms) found by whole-exome -sequencing in at least two affected children of the trios (T). Chr., chromosome; SNP, single nucleotide polymorphism; QUAL., a quality parameter measuring the probability p that the observation of the variant is due to chance (for ex: QUAL = n, p = 1/n). As detailed in the text, Alamut® Visual integrates missense variant pathogenicity prediction tools and in silico study of variants’ effect on RNA splicing, allowing the assessment of their potential impact on splice junctions and splicing regulatory sequences. Alamut® Visual helped us also to exclude well known mutations identified in recessive diseases for those genes which have been related to known genetic diseases (as shown in Table 3). (DOCX 23 kb) [file 12920_2018_338_MOESM1_ESM.docx]

| **T1+T2** | 9 | 139368953 | SNP | 28526 | 602 | **SEC16A** | c.3115C>T  p.Arg1039Cys  (NM_014866.1) | rs3812594 | 0.14 | **0.689 *** | Class 3  Unknown  Exon skip? | 0.21 |
| --- | --- | --- | --- | --- | --- | --- | --- | --- | --- | --- | --- | --- |
| **T1+T2** | 5 | 89985882 | SNP | 28610 | 705 | **ADGRV1** | c.6695A>G  p.Tyr2232Cys  (NM_032119.3) | rs10037067 | **0.01 *** | **0926 *** | Class 3  Unknown  Exon skip? | 0.39 |
|  |  |  |  |  |  |  |  |  |  |  |  |  |
| **T1+T3** | 1 | 39352271 | SNP | 24599  39683 | 564  515 | **RHBDL2** | c.1057C>A  p.Leu353Met  (NM_001304746.1) | rs2147914 | 0.16 | **0.730 *** | Class 3  Unknown | 0.39 |
| **T1+T3** | 2 | 185802211 | In frame DEL | 15197  27219 | 214  199 | **ZNF804A** | c.2090_2092dupCAA  p.Thr697dup  (NM_194250.1) | rs3046266 | Probably damaging | Probably damaging | Class 3  Unknown | NR |
| **T1+T3** | 11 | 65547333 | SNP | 15898  27369 | 187  219 | **AP5B1** | c.631C>T  p.Leu211Phe  (NM_138368.4) | rs12146493 | **0 *** | **0.999 *** | Class 3  Exon skip | 0.29 |
| **T1+T3** | 11 | 88911696 | SNP | 57682  88152 | 994  723 | **TYR** | c.575C>A  p.Ser192Tyr  (NM_000372.4) | rs1042602 | **0.01 *** | **0.964 *** | Class 3  Unknown | 0.25 |
| **T1+T3** | 19 | 17025292 | SNP | 4364  11624 | 186  186 | **CPAMD8** | c.3944C>T  p.Thr1315Ile  (NM_015692.2) | rs706761 | 0.08 | **0.637 *** | Class 3  Unknown | 0.43 |
|  |  |  |  |  |  |  |  |  |  |  |  |  |
| **T2+T3** | 4 | 152201053 | SNP | 16019  22456 | 245  327 | **PRSS48** | c.158G>A  p.Cys53Tyr  (NM_183375.2) | rs36097019 | **0 *** | **1 *** | Class 3  Unknown | 0.34 |
|  |  |  |  |  |  |  |  |  |  |  |  |  |
| **T1+T2+T3** | 14 | 20666175 | FRAME  SHIFT | 95633  114461  129852 | 1012  804  539 | **OR11G2** | c.687dupA  p.Gly230fs  (NM_001005503.1) | rs55781225 | Probably damaging | Probably damaging | Class 3  Unknown  Frameshift | 0.32 |

**Table S1**
