# Supplementary material for: Whole exome sequencing in three families segregating a pediatric case of sarcoidosis
Source: BMC Med Genomics. 2018 Mar 6;11:23. doi: 10.1186/s12920-018-0338-x (PMC5839022; doi:10.1186/s12920-018-0338-x)
Supplement: Supplementary file 2 — Table S2. Recessive variants shared by a common gene in at least two different trios. Possibly pathogenic recessive variants observed at different positions for a single gene in at least two affected children of the trios (T). Abbreviations are the same as in Tables 1, 2 and Additional file 1: Table S1. (DOCX 31 kb) [file 12920_2018_338_MOESM2_ESM.docx]

| **OBSCN** | T1 | 1 | 228505204 | SNP | 11933 | 256 | c.16472G>A  p.Arg5491His  (NM_001271223.2) | rs4653942 | **0 *** | **0.91 *** | Class 3  Unknown | 0.26 |
| --- | --- | --- | --- | --- | --- | --- | --- | --- | --- | --- | --- | --- |
|  | T3 | 1 | 228494790 | SNP | 39728 | 479 | c.14986G>A  p.Gly4996Arg  (NM_001271223.2) | rs435776 | **0.01 *** | **0.939 *** | Class 3  Unknown | 0.41 |
| **ASPN** | T1 | 2 | 95237024 | I.F. INS | 12154 | 318 | c.150_152dupTGA  **p.Asp51ter**  (NM_017680.4) | rs3078372 | **In frame INS *** | **In frame INS *** | Class 4  Likely pathogenic | nd |
|  | T2 | 2 | 95237024 | I.F. DEL | 20429 | 212 | c.150_152delTGA  p.Asp51del  (NM_017680.4) | rs397838876 | **In frame**  **DEL *** | **In frame**  **DEL *** | Class 3  Unknown | nd |
| **WFDC3** | T1 | 20 | 44417593 | SNP | 9457 | 361 | c.188G>T  p.Arg63Leu  (NM_080614.1) | rs73122754 | 0.06 | **0.716 *** | Class 3  Unknown  Exon skip? | 0.045 |
|  | T2 | 20 | 44417675 | SNP | 30629 | 181 | c.106C>G  p.His36Asp  (NM_080614.1) | rs6032538 | **0.04 *** | 0 | Class 3  Unknown | 0.31 |
| **SLC16A8** | T1 | 22 | 38477342 | SNP | 14719 | 168 | c.703A>T  p.Arg235Trp  NM_013356.2 | rs4289289 | **0.02 *** | **0.84 *** | Class 3  Unknown  Exon skip? | 0.26 |
|  | T3 | 22 | 38474696 | SNP | 56317 | 471 | c.1214T>C  p.Val405Ala  NM_013356.2 | rs2076371 | 1 | 0.001 | Class 3  Unknown | 0.41 |
| **CCT6B** | T1 | 17 | 33286664 | SNP | 24463 | 685 | c.143T>C  p.Val48Ala  (NM_006584.3) | rs2230552 | **0.02 *** | **0.95 *** | Class 3  Unknown | 0.25 |
|  | T2 | 17 | 33288363 | SNP | 26757 | 131 | c.50G>A  p.Arg17Gln  (NM_006584.3) | rs9635769 | 0.92 | 0 | Class 3  Unknown | 0.44 |
| **AFAP1** | T2 | 4 | 7780582 | SNP | 19706 | 215 | c.1804G>A  p.Val602Met  (NM_001134647.1) | rs41264705 | 0.13 | 0.018 | Class 3  Unknown | 0.22 |
|  | T3 | 4 | 7802227 | SNP | 13612 | 262 | c.1208C>G  p.Ser403Cys  (NM_001134647.1) | rs28406288 | **0 *** | **0.852 *** | Class 3  Unknown  Exon skip? | 0.14 |
| **HSD17B4** | T2 | 5 | 118860938 | SNP | 4665 | 183 | c.1606T>C  p.Trp536Arg  NM_001199291.2 | rs11539471 | 0.26 | 0.024 | Class 3  Unknown | 0.089 |
|  | T3 | 5 | 118811533 | SNP | 17886 | 386 | c.392G>A  p.Arg131His  (NM_001199291.2) | rs25640 | **0.01 *** | **0.71 *** | Class 3  Unknown | 0.44 |
|  |  |  |  |  |  |  |  |  |  |  |  |  |
| **DNAH11** | T2 | 7 | 21924014 | SNP | 37644 | 377 | c.12493A>G  p.Met4165Val  (NM_001277115.1) | rs6461613 | 0.19 | 0.019 | Class 3  Unknown | 0.24 |
|  | T3 | 7 | 21582963 | SNP | 21837 | 95 | c.100G>T  **p.Glu34ter**  (NM_001277115.1) | rs2285943 | **STOP *** | **STOP *** | Class 3  Unknown  Exon skip? | 0.49 |
| **CNGB1** | T2 | 16 | 57949224 | SNP | 8350 | 202 | c.2233C>A  p.Leu745Ile  (NM_001297.4) | rs10459809 | 0.14 | **0.298*** | Class 3  Unknown  Exon skip? | 0.13 |
|  | T3 | 16 | 57996960 | SNP | 14257 | 225 | c.299G>A  p.Arg100His  (NM_001297.4) | rs13336595 | **0.01 *** | **0.519 *** | Class 3  Unknown | 0.22 |
| **MARCH10** | T2 | 17 | 60824224 | SPLICE | 2814 | 138 | c.496+2T>C  SPLICE DONOR  (NM_001288779.1) | rs17746153 | **SPLICE**  **DONOR** | **SPLICE**  **DONOR** | Class 3  Unknown  SPLICE DEFECT | 0.26 |
|  | T3 | 17 | 60814273 | SNP | 40915 | 370 | c.1070T>C  p.Phe357Ser  (NM_001288779.1) | rs9891498 | **0 *** | **0.971 *** | Class 3  Unknown | 0.36 |
| **RHBG** | T1 | 1 | 156347131 | SNP | 33609 | 360 | c.227G>A  p.Gly76Asp  NM_020407.4 | rs2245623 | **0 *** | **0.971 *** | Class 3  Unknown  Exon skip? | 0.37 |
|  | T2 | 1 | 156347834 | SNP | 16898 | 201 | c.428C>T  p.Val143Asp  NM_020407.4 | rs11586833 | **0 *** | **0.795 *** | Class 3  Unknown | 0.26 |
|  | T3 | 1 | 156351699 | SNP | 36239 | 374 | c.943C>T  p.Gly315Arg  NM_020407.4 | rs3748569 | **0 *** | **0.996 *** | Class 3  Unknown | 0.48 |
| **KNL1**  **(CASC5)** | T1 | 15 | 40916237 | SNP | 57541 | 697 | c.3853A>G  p.Lys1285Glu  (NM_170589.4) | rs17747633 | **0.05 *** | **0.058** | Class 3  Unknown  Exon skip? | 0.32 |
|  | T2 | 15 | 40915190 | SNP | 183916 | 368 | c.2806A>G  p.Arg936Gly  NM_170589.4 | rs8040502 | 1 | 0 | Class 3  Unknown | 0.24 |
|  | T3 | 15 | 40914177 | SNP | 66997 | 297 | c.1793T>C  p.Met598Thr  NM_170589.4 | rs11858113 | **0.01 *** | 0.006 | Class 3  Unknown | 0.39 |

**Table S2**
