# Supplementary material for: Whole exome sequencing in three families segregating a pediatric case of sarcoidosis
Source: BMC Med Genomics. 2018 Mar 6;11:23. doi: 10.1186/s12920-018-0338-x (PMC5839022; doi:10.1186/s12920-018-0338-x)
Supplement: Supplementary file 3 — Table S3. Composite heterozygocity observed in a common gene in at least two different trios. Possibly pathogenic compound heterozygous variants (allelic heterogeneity) observed in different positions of a common gene in at least two trios. The origin of either the paternal and maternal allele was detailed for each variant. Abbreviations are the same as in Tables 1, 2, Additional files 1 and 2: Tables S1 and S2. (DOCX 51 kb) [file 12920_2018_338_MOESM3_ESM.docx]

| **CMYA5** | **T1** | 5 | 79086883 | SNP | Paternal | 6368 | 443 | c.440G>A  p.Arg147Gln  (NM_153610.4) | rs62621858 | **0.02 *** | **0.602 *** | 0.007 |
| --- | --- | --- | --- | --- | --- | --- | --- | --- | --- | --- | --- | --- |
|  | **T1** | 5 | 79028513 | SNP | Maternal | 10158 | 586 | c.3925A>G  p.Ile1309Val  (NM_153610.4) | rs16877133 | 0.18 | **0.785 *** | 0.18 |
|  | **T2** | 5 | 79029594 | SNP | Paternal | 11912 | 314 | c.5006T>C  p.Leu1669Ser  (NM_153610.4) | rs1019762 | 0.1 | 0.008 | 0.14 |
|  | **T2** | 5 | 79095299 | SNP | Maternal | 2388 | 216 | c.12070A>G  p.Asn4024Asp  (NM_153610.4) | rs115059007 | **0 *** | **0.999 *** | 0.017 |
|  |  |  |  |  |  |  |  |  |  |  |  |  |
| **PCDHB16** | **T1** | 5 | 140564221 | SNP | Paternal | 4099 | 360 | c.2087C>T  p.Ser696Leu  (NM_020957.3) | rs138310875 | **0.01 *** | 0.117 | 0.00002 |
|  | **T1** | 5 | 140563173 | SNP | Maternal | 33839 | 564 | c.1039G>T  p.Val347Leu  (NM_020957.3) | rs28664170 | 1 | 0.003 | 0.153 |
|  | **T2** | 5 | 140563173 | SNP | Paternal | 42768 | 440 | c.1039G>T  p.Val347Leu  (NM_020957.3) | rs28664170 | 1 | 0.003 | 0.153 |
|  | **T2** | 5 | 140562739 | SNP | Maternal | 4329 | 270 | c.605G>A  p.Arg202Gln  NM_020957.3 | rs61743469 | **0.01 *** | **0.623 *** | 0.042 |
|  |  |  |  |  |  |  |  |  |  |  |  |  |
| **RREB1** | **T1** | 6 | 7231843 | SNP | Paternal | 2748 | 226 | c.3511G>A  p.Asp1171Asn  (NM_001003698.3) | rs9379084 | **0 *** | **0.999 *** | 0.11 |
|  | **T1** | 6 | 7246998 | SNP | Maternal | 6191 | 202 | c.4150G>A  p.Gly1384Arg  NM_001003698.3 | rs2281833 | 0.12 | 0 | 0.27 |
|  | **T2** | 6 | 7230680 | SNP | Paternal | 33331 | 371 | c.2348G>T  p.Gly783Val  NM_001003698.3 | rs9502564 | 0.06 | 0.156 | 0.44 |
|  | **T2** | 6 | 7247344 | SNP | Maternal | 14853 | 173 | c.4496C>A  p.Ser1499Tyr  NM_001003698.3 | rs35742417 | **0.01 *** | 0.025 | 0.14 |
|  |  |  |  |  |  |  |  |  |  |  |  |  |

| **AIM1L** | **T1** | 1 | 26670781 | SNP | Paternal | 5375 | 398 | c.2368C>T  p.Arg790Cys  (NM_001039775.3) | rs150982279 | **0 *** | **0.999 *** | 0.033 |
| --- | --- | --- | --- | --- | --- | --- | --- | --- | --- | --- | --- | --- |
|  | **T1** | 1 | 26663362 | SNP | Maternal | 18207 | 89 | c.3881G>A  p.Ser1294Asn  (NM_001039775.3) | rs10751735 | 0.31 | 0.03 | 0.44 |
|  | **T3** | 1 | 26670650 | I.F. INS | Paternal | 9506 | 330 | c.2496_2498dupGGA  p.Glu833dup  (NM_001039775.3) | rs199784730 | IN FRAME  INSERTION | IN FRAME  INSERTION | 0.0043 |
|  | **T3** | 1 | 26663362 | SNP | Maternal | 23201 | 113 | c.3881G>A  p.Ser1294Asn  (NM_001039775.3) | rs10751735 | 0.31 | 0.03 | 0.44 |
|  |  |  |  |  |  |  |  |  |  |  |  |  |
| **IDO2** | T1 | 8 | 39862881 | SNP | Paternal | 14321 | 289 | c.742C>T  p.Arg248Trp  (NM_194294.2) | rs10109853 | **0 *** | **1 *** | 0.48 |
|  | T1 | 8 | 39873053 | SNP | Maternal | 7771 | 184 | c.1195G>A  p.Ala399Thr  (NM_194294.2) | rs72632016 | 0.57 | 0.024 | 0.06 |
|  | T3 | 8 | 39872935 | SNP | Paternal | 21240 | 451 | c.1077T>A  p.Tyr359*  (NM_194294.2) | rs4503083 | **STOP *** | **STOP *** | 0.226 |
|  | T3 | 8 | 39862893 | SNP | Maternal | 3510 | 154 | c.754T>A  p.Ser252Thr  (NM_194294.2) | rs35212142 | **0.02 *** | **0.444 *** | 0.02 |
|  |  |  |  |  |  |  |  |  |  |  |  |  |
| **KIR3DL1** | T1 | 19 | 55331308 | SNP | Paternal | 6349 | 467 | c.496C>A  p.Arg166Ser  (NM_013289.2) | rs1065331 | 0.13 | 0.022 | 0.028 |
|  | T1 | 19 | 55333275 | SNP | Maternal | 31813 | 544 | c.911G>T  p.Trp304Leu  (NM_013289.2) | rs35974949 | **0.01 *** | **0.999 *** | 0.39 |
|  | T3 | 19 | 55333139 | SNP | Paternal | 8048 | 425 | c.775G>C  p.Gly259Arg  (NM_013289.2) | rs1049215 | **0.02 *** | 0.371 | 0.13 |
|  | T3 | 19 | 55333275 | SNP | Maternal | 38415 | 407 | c.911G>T  p.Trp304Leu  (NM_013289.2) | rs35974949 | **0.01 *** | **0.999 *** | 0.39 |
|  |  |  |  |  |  |  |  |  |  |  |  |  |

| **TDRD5** | T2 | 1 | 179562792 | SNP | Paternal | 11540 | 337 | c.430G>A  p.Ala144Thr  (NM_001199085.1) | rs77758448 | 0.15 | 0.075 | 0.029 |
| --- | --- | --- | --- | --- | --- | --- | --- | --- | --- | --- | --- | --- |
|  | T2 | 1 | 179631242 | SNP | Maternal | 3020 | 100 | c.2326G>A  p.Glu776Lys  (NM_001199085.1) | rs35448215 | **0.01 *** | 0.068 | 0.198 |
|  | T3 | 1 | 179600001 | SNP | Paternal | 18343 | 413 | c.1072A>G  p.Lys358Glu  (NM_001199085.1) | rs6704505 | 1 | 0 | 0.057 |
|  | T3 | 1 | 179631242 | SNP | Maternal | 5029 | 112 | c.2326G>A  p.Glu776Lys  (NM_001199085.1) | rs35448215 | **0.01 *** | 0.068 | 0.198 |
|  |  |  |  |  |  |  |  |  |  |  |  |  |
| **LPA** | T2 | 6 | 161007538 | SNP | Paternal | 28059 | 303 | c.4072C>G  p.Leu1358Val  (NM_005577.2) | rs7765803 | 1 | 0 | 0.36 |
|  | T2 | 6 | 160966559 | SNP | Maternal | 2959 | 262 | c.5311C>T  p.Arg1771Cys  (NM_005577.2) | rs139145675 | **0 *** | **0.95 *** | 0.0019 |
|  | T3 | 6 | 160952816 | SNP | Paternal | 9865 | 368 | c.6068A>G  p.Tyr2023Cys  (NM_005577.2) | rs41267807 | **0 *** | **0.586 *** | 0.01 |
|  | T3 | 6 | 161006172 | SNP | Maternal | 7104 | 423 | c.4195A>C  p.Thr1399Pro  (NM_005577.2) | rs41272110 | **0.04 *** | **0.99 *** | 0.11 |
|  |  |  |  |  |  |  |  |  |  |  |  |  |
| **DNAH11** | T2 | 7 | 21893993 | SNP | Paternal | 46459 | 445 | c.11122G>T  p.Val3708Leu  (NM_001277115.1) | rs4722064 | **0.02 *** | **0.611 *** | 0.43 |
|  | T2 | 7 | 21584693 | SNP | Maternal | 1610 | 159 | c.421G>T  p.Asp141Tyr  (NM_001277115.1) | rs72655969 | **0 *** | **0.601 *** | 0.012 |
|  | T3 | 7 | 21628242 | SNP | Paternal | 20259 | 553 | c.1961C>G  p.Ser654Cys  (NM_001277115.1) | rs62441683 | 0.67 | 0.02 | 0.13 |
|  | T3 | 7 | 21678643 | SNP | Maternal | 19583 | 850 | c.4904A>G  p.Asp1635Gly  (NM_001277115.1) | rs17144835 | **0 *** | **0.783 *** | 0.045 |

| **PIEZO1** | T2 | 16 | 88789666 | I.F INS | Paternal | 7897 | 93 | c.4400_4405dupAGCAGG  p.Glu1467_Gln1468dup  (NM_001142864.3) | rs11281795 | IN FRAME  INS | IN FRAME  INS | 0.11 |
| --- | --- | --- | --- | --- | --- | --- | --- | --- | --- | --- | --- | --- |
|  | T2 | 16 | 88787607 | I.F DEL | Maternal | 17528 | 259 | c.5632_5634delAAG  p.Lys1878del  (NM_001142864.3) | rs150376294 | IN FRAME  DEL | IN FRAME  DEL | 0.35 |
|  | T3 | 16 | 88783618 | SNP | Paternal | 12798 | 584 | c.6473A>G  p.Lys2158Arg  (NM_001142864.3) | rs200506892 | 0.52 | **0.766 *** | 0.0003 |
|  | T3 | 16 | 88789046 | SNP | Maternal | 3431 | 156 | c.4720G>C  p.Glu1574Gln  (NM_001142864.3) | rs773645030 | 0.21 | 0.257 | 0.00006 |
|  |  |  |  |  |  |  |  |  |  |  |  |  |
| **FCGPB** | T2 | 19 | 40421537 | SNP | Paternal | 2396 | 208 | c.2384C>T  p.Pro795Leu  (NM_003890.2) | rs147566832 | **0.03 *** | 0.041 | 0.0185 |
|  | T2 | 19 | 40392588 | SNP | Maternal | 1489 | 88 | c.7916G>A  p.Ser2639Asn  (NM_003890.2) | rs62108885 | 0.42 | 0.016 | 0.16 |
|  | T3 | 19 | 40408821 | SNP | Paternal | 30836 | 334 | c.4018G>C  p.Val1340Leu  (NM_003890.2) | rs11083543 | 0.4 | **0.559*** | 0.258 |
|  | T3 | 19 | 40412179 | SNP | Maternal | 3948 | 266 | c.3449C>T  p.Pro1150Leu  (NM_003890.2) | rs150293178 | **0.03 *** | 0.056 | 0.0006 |
|  |  |  |  |  |  |  |  |  |  |  |  |  |
| **PCNT** | T2 | 21 | 47808679 | SNP | Paternal | 4964 | 233 | c.3487C>T  p.Arg1163Cys  (NM_006031.5) | rs7279204 | **0 *** | **0.876 *** | 0.15 |
|  | T2 | 21 | 47821588 | SNP | Maternal | 36914 | 350 | c.4915A>G  p.Ile1639Val  (NM_006031.5) | rs6518291 | 1 | 0 | 0.25 |
|  | T3 | 21 | 47817247 | SNP | Paternal | 10997 | 610 | c.4285C>T  p.Arg1429Cys  NM_006031.5 | rs62224222 | 0.1 | **0.876 *** | 0.0037 |
|  | T3 | 21 | 47836403 | SNP | Maternal | 17574 | 257 | c.6571T>C  p.Ser2191Pro  NM_006031.5 | rs34151633 | 0.26 | **0.665 *** | 0.036 |

**Table S3**
