# Supplementary material for: Whole exome sequencing in three families segregating a pediatric case of sarcoidosis
Source: BMC Med Genomics. 2018 Mar 6;11:23. doi: 10.1186/s12920-018-0338-x (PMC5839022; doi:10.1186/s12920-018-0338-x)
Supplement: Supplementary file 4 — Figure S1. CADD scoring of prioritized variants versus other variants in the selected genes. (PDF 71 kb) [file 12920_2018_338_MOESM4_ESM.pdf]

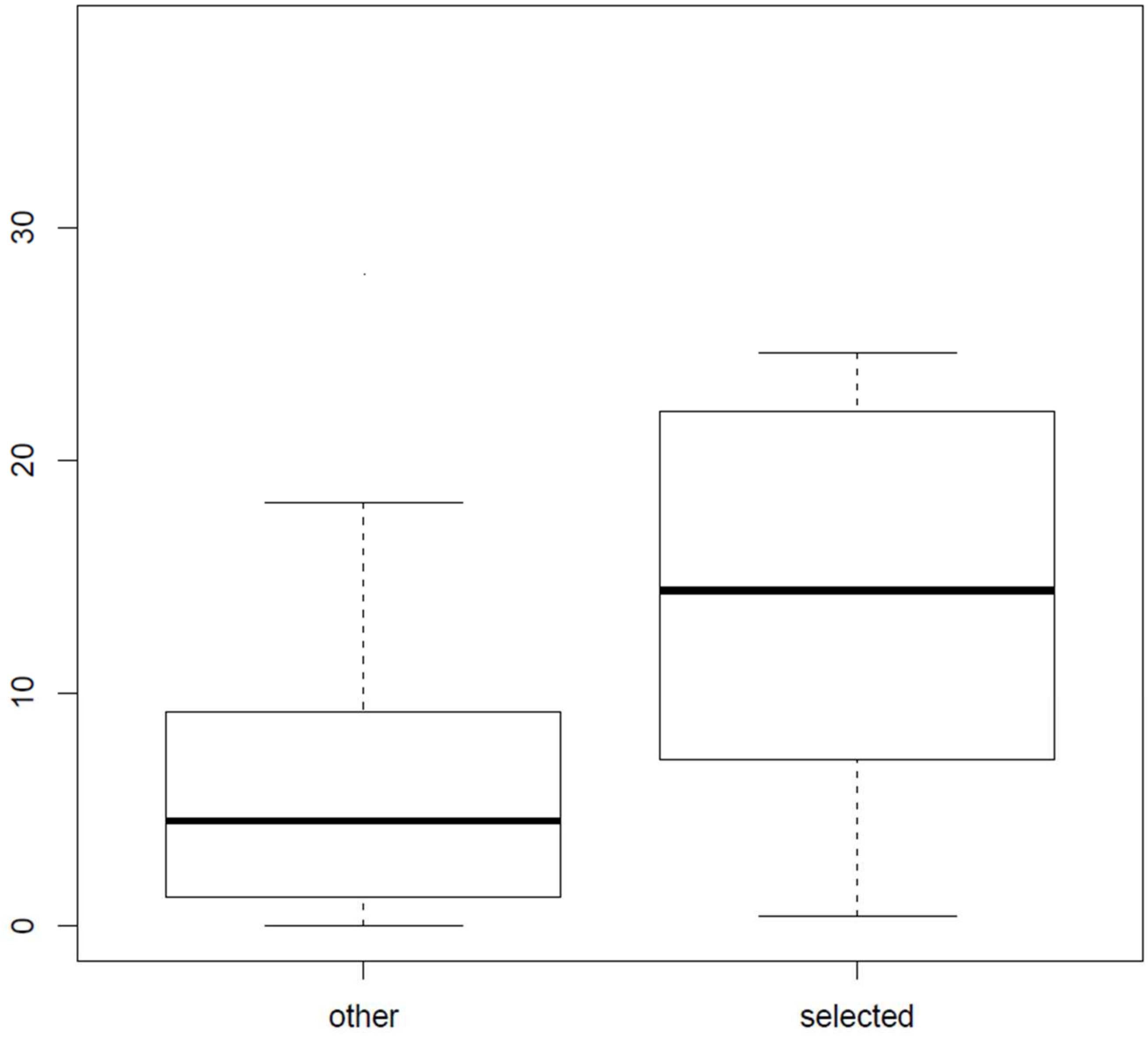

ADDITIONNAL FILE 1 – Figure S1 - CADD scoring of prioritized variants versus other variants in the selected genes
